# The Characterization of Surface Acoustic Wave Devices Based on AlN-Metal Structures

**DOI:** 10.3390/s16040526

**Published:** 2016-04-12

**Authors:** Lin Shu, Bin Peng, Chuan Li, Dongdong Gong, Zhengbing Yang, Xingzhao Liu, Wanli Zhang

**Affiliations:** 1 State Key Laboratory of Electronic Thin Films and Integrated Devices, University of Electronic Science and Technology of China, Chengdu 610054, China; s89s89s@126.com (L.S.); uestc_lich@hotmail.com (C.L.); 15196609270@163.com (D.G.); xzliu@uestc.edu.cn (X.L.); wlzhang@uestc.edu.cn (W.Z.); 2 China Gas Turbine Establishment, Jiangyou 621703, China; zbyang668@163.com

**Keywords:** AlN film, TC4, surface acoustic wave, layered structure, simulation

## Abstract

We report in this paper on the study of surface acoustic wave (SAW) resonators based on an AlN/titanium alloy (TC4) structure. The AlN/TC4 structure with different thicknesses of AlN films was simulated, and the acoustic propagating modes were discussed. Based on the simulation results, interdigital transducers with a periodic length of 24 μm were patterned by lift-off photolithography techniques on the AlN films/TC4 structure, while the AlN film thickness was in the range 1.5–3.5 μm. The device performances in terms of quality factor (Q-factor) and electromechanical coupling coefficient (*k*^2^) were determined from the measure *S*_11_ parameters. The Q-factor and *k*^2^ were strongly dependent not only on the normalized AlN film thickness but also on the full-width at half-maximum (FWHM) of AlN (002) peak. The dispersion curve of the SAW phase velocity was analyzed, and the experimental results showed a good agreement with simulations. The temperature behaviors of the devices were also presented and discussed. The prepared SAW resonators based on AlN/TC4 structure have potential applications in integrated micromechanical sensing systems.

## 1. Introduction

In recent years, there have been growing demands for surface acoustic waves (SAWs) strain sensors in structural health monitoring (SHM) due to its potential applications in wireless and passive measurements. Most SAW strain sensors are prepared with piezoelectric crystal substrates such as quartz, langasite (LGS, La_3_Ga_5_SiO_14_), lithium niobate (LiNbO_3_), and zinc oxide (ZnO) [1]. For example, SAW orthogonal frequency coded (OFC) strain sensors using a LGS substrate were researched by Wilson [2]. Furthermore, another strain sensor based on a one-port SAW resonator using quartz was investigated by Stoney [3]. Yet constraints such as mass, volume, and bonding techniques often limit the usage of SHM sensors in practical applications. The SAW strain sensors must be pasted onto the measured components with adhesives. This would increase the measurement error and have a risk of peeling off in harsh environment.

An AlN film SAW sensor integrated with a metal structure has been demonstrated in our previous work [4]. A layer of AlN film was directly sputtered onto the metal substrate, and a SAW resonator was fabricated on the AlN film. Compared with conventional SAW sensors, the SAW sensors in this work can be fabricated directly on the components without any adhesives. This would decrease the measurement error caused by the adhesives in a harsh environment. Moreover, because the thickness of AlN film is far less than the wavelength of the acoustic waves, the acoustic waves penetrate the underlying substrate. In this case, the properties of the acoustic waves are mainly determined by the substrates and the AlN films. Hence, compared with conventional SAW sensors, the AlN film SAW devices integrated with a metal structure would be more sensitive to the mechanical deformation of the metal substrates [4].

In this work, we report on the design, simulation, and fabrication of the AlN film SAW devices integrated with metal components systematically. The dependence of the acoustic velocity and the electromechanical coupling coefficient of the SAW devices on the AlN film thickness are presented and discussed. Lastly, the temperature behaviors of the devices with different AlN film thicknesses are presented and discussed.

## 2. Design and Simulation

**Figure 1:**
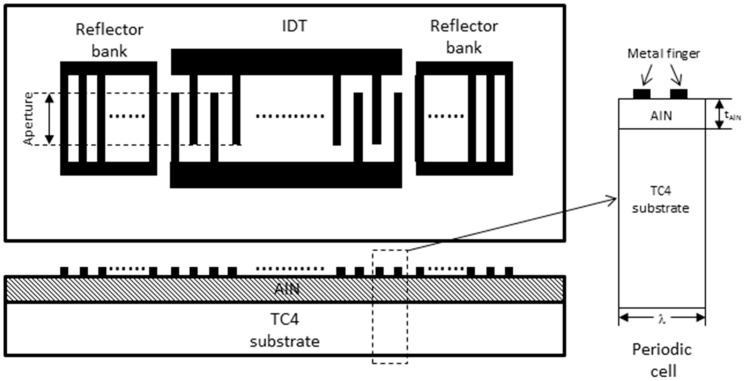
Schematic illustration of the one-port surface acoustic wave (SAW) resonator and the modeling periodic cell.

In this work, we designed a one-port SAW resonator, which consists of an interdigital transducer (IDT) and two reflector banks. The IDT contained 101 equal-interval-finger electrodes, and each reflector bank contained 400 short-circuited gratings. The finger width of the IDTs was 6 μm, yielding an acoustic wavelength (λ) of 24 μm. The acoustic aperture W was 100λ. The IDT was patterned on the AlN films, which were deposited on a TC4 (titanium alloy, known as Ti-6Al-4V) alloy substrate. The schematic illustration of the one-port SAW resonator is presented in Figure 1. Because the IDTs are periodic in nature, one period cell of the IDT electrode is sufficient to model the SAW resonator as a whole. The height of the simulation cell only extends a few wavelengths down to the bottom of the substrate, because the SAW has almost died out at the lower boundary. Since the length of the electrode is far larger than its width, edge effects of the electrodes can be ignored, and the model geometry can be reduced to a periodic cell [5]. The geometry of the SAW structure used in the simulation is shown in Figure 1.

We analyzed the acoustic wave characteristics in the AlN/TC4 structure using COMSOL software to determine the velocity and electromechanical coupling coefficient (*k*^2^) for the acoustic waves. The material constants of AlN and TC4 are listed in Table 1.

The mode of acoustic wave was identified with an Eigen mode simulation; thus, the acoustic wave velocity was calculated by

**Figure 2:**
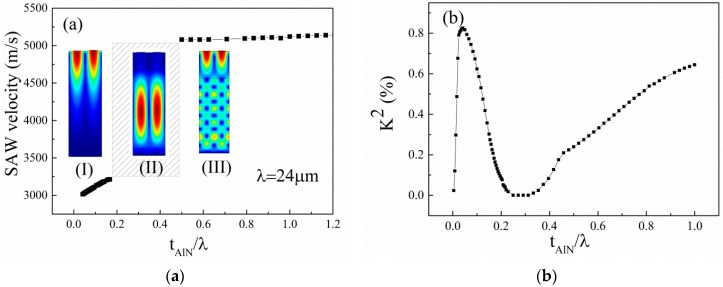
(**a**) Dependence of the simulated acoustic wave velocity on the normalized thickness of the AlN film and the typical mode schematic diagram of the acoustic waves with different thicknesses of the AlN film. The color corresponds to the displacement amplitude; (**b**) Dependence of the simulated electromechanical coupling coefficient of the devices on the normalized thickness of the AlN film.

(1) v=λf

where λ is the wavelength of the acoustic wave propagation, and *f* is the Eigen-frequency result from the simulation. The calculated acoustic wave velocity as a function of the normalized thickness of the AlN film (*t_AlN_*/λ) is shown in Figure 2a. Here, the *t_AlN_* is the thickness of the AlN films. The typical simulation diagrams with different AlN film thicknesses are also presented in Figure 2a. The electromechanical coupling coefficient *k*^2^ for the AlN/TC4 structure is calculated by [8]:

(2) $$k^{2}=2\times \frac{v_{0}-v_{m}}{v_{0}}$$

where the *v*_0_ and *v_m_* are phase velocities, when the electrical boundary conditions of the AlN surface are assumed to be electrically free and shorted, respectively. The simulated *k*^2^ is shown in Figure 2b.

From Figure 2a, it can be found that different acoustic wave propagation modes occur in the AlN/TC4 bilayer with different AlN film thicknesses. In Figure 2a, we observe that the phase velocity is dispersive; that is, it is dependent on the normalized film thickness [9]. There are three regions, which are marked as (I), (II), and (III). In region (I), because the *t_AlN_*/λ is very small, the particle displacements extend far into the substrate, causing the phase velocity to approach the value in bare substrate. The acoustic wave velocity in region (I) is in the range 3000–3200 m/s, which is very close to the acoustic wave velocity in Ti (2958 m/s [7]). This suggests that the acoustic wave is excited in AlN film and mainly propagates in the TC4 substrate in region (I). In region (III), the motion of particles is mainly concerned in the vicinity of AlN film, causing the acoustic wave velocity to approach the value in the layered AlN film. Both the Rayleigh acoustic wave and the leaky acoustic wave exist when the *t_AlN_* is comparable to *λ*. With a further increase in AlN film thickness, the leaky wave disappears, and only the Rayleigh SAW occurs in the thick AlN film. The acoustic wave velocity is about 5100–5200 m/s in region (III), which is close to the acoustic wave velocity in AlN film (about 5100–5600 m/s [10,11]). This result confirms that the acoustic wave mainly propagates in the AlN film and barely scatters into the TC4 substrate in region (III). These conclusions also explain why the SAW velocity increases with an increasing *t_AlN_*/λ in regions (I) and (III). In addition, we find that the Rayleigh wave cannot be excited when 0.2 < *t_AlN_*/λ < 0.5, corresponding to region (II). We think this is due to the low *k*^2^ value in region (II) related to the specific AlN/TC4 layered structure, as shown in Figure 2b. In Figure 2b, the *k*^2^ increases firstly with an increase of *t_AlN_*/λ and reaches the relative maximum value of 0.81% when the *t_AlN_*/λ is about 0.08%. The *k*^2^ decreases with the further increase of *t_AlN_*/λ. The maximum *k*^2^ of the layered structure is influenced by electrical boundary conditions and the material constant [12]. In our simulation model, the interface between the AlN film and the metal substrate is electrically shorted, which leads to a large *k*^2^ relative to the open electric conditions of the interface. We can find that the *k*^2^ approaches 0 when the AlN film thickness is close to 0. This is because the piezoelectricity of the system in fact disappears. Thus, electrical boundary conditions on the surface do not influence the mode of the acoustic wave. These results are similar with the results reported in [12].

The propagation loss for the layered acoustic devices can be calculated by [13]:

(3) $$\alpha =\frac{8.686\pi f_{r}}{Qf_{g}}$$

(4) $$f_{g}=v_{g}/\lambda$$

(5) $$v_{g}=\frac{\partial \omega }{\partial k}=V_{p}+k\frac{dV_{p}}{dk}$$

where *f_r_* is the resonance frequency, *Q* is the quality factor, *v_g_* is the group velocity of the SAW, *v_p_* is the phase velocity of the SAW, and k is the relative wave number of the SAW, which can be obtained by *k* = 2π*t_AlN_/λ.

**Figure 3:**
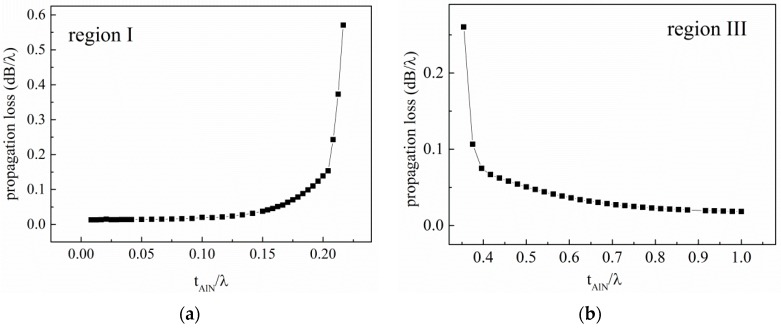
(**a**) The simulated propagation loss in region (I); (**b**) The propagation loss in region (III).

In the calculation, the *Q* is obtained by calculating the admittance of the layered structure. The admittance Y of the device can be calculated by [5]:

(6) $$Y=j\omega Q_{i}/V_{i}$$

where *ω* is the angular frequency, *Q_i_* is the complex charge in the electrodes, and *V_i_* is the potential. The calculated propagation loss in regions (I) and (III) is presented in Figure 3.

## 3. Fabrication

**Figure 4:**
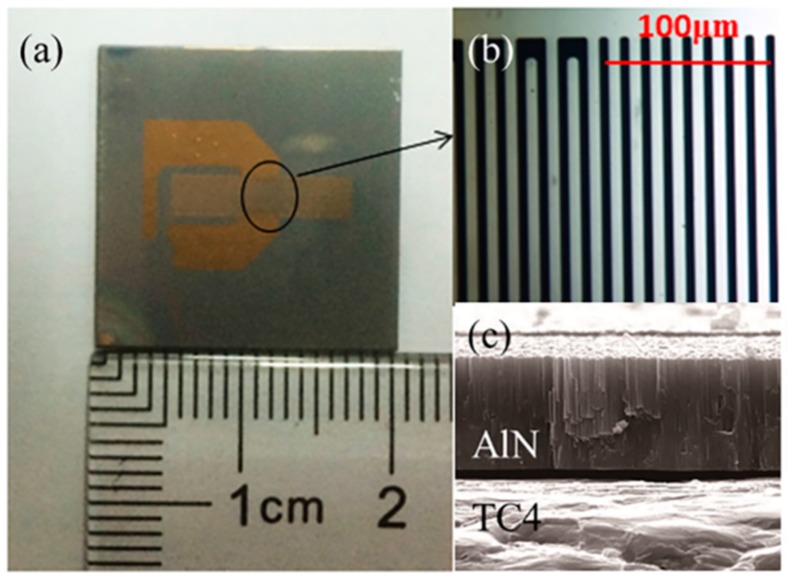
(**a**) The photograph of the SAW devices deposited onto AlN/TC4 bilayer; (**b**) The detailed picture of the electrodes; (**c**) The cross-section SEM of the AlN/TC4 structure.

We fabricated the SAW devices with the AlN/TC4 structure when *t_AlN_*/λ < 0.2. The thin AlN films were prepared via middle-frequency magnetron sputtering on the TC4 substrate. The dimension of the substrate was 20 mm × 20 mm × 0.8 mm, and all of the substrates were mechanically polished before sputtering. A two-step deposition process was used to deposit AlN films onto the TC4 substrate. The growth process is studied in [14] in detail. By controlling the deposition time, the thickness of the AlN film was adjusted from 1.5 μm to 3.5 μm, yielding a *t_AlN_*/λ from 0.0625 to 0.1458. On the top of the AlN films, a one-port SAW resonator was patterned via lift-off photolithography techniques. The electrodes consisted of a 10-nm-thick Ti adhesion layer and a 100-nm-thick Au film. Photos of the devices are shown in Figure 4.

The crystal structures of the AlN films were characterized by *X*-ray diffraction (XRD) (Cu-Kα, Bede-D1). The degree of c-axis orientation of the AlN films was characterized by the full width at half maximum (FWHM) of the AlN (002) diffraction peak. The characterization of the SAW resonator was performed by measuring *S*_11_ parameters as a function of frequency using a vector network analyzer (VNA, Agilent E5071b, Agilent Technologies Inc., Santa Clara, CA, USA) and a microwave micro-prober.

## 4. Results and Discussion

**Figure 5:**
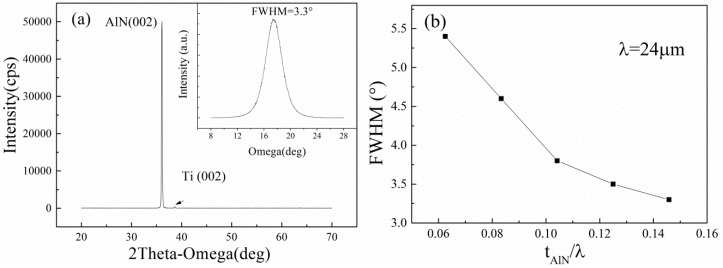
(**a**) X-ray diffraction (XRD) results of the 3.5 μm thick AlN film. Inset: AlN (002) peak rocking curve of AlN films; (**b**) Dependence of full width at half maximum (FWHM) values of the AlN films on the normalized thickness of AlN films.

Typical XRD-spectra of the AlN films on the TC4 substrate is presented in Figure 5a. The thickness of the AlN film is 3.5 μm, corresponding to *t_AlN_*/λ of 0.1458. The FWHM value of the *X*-ray rocking curve for the (002) oriented thin AlN film is only 3.3°, which indicates that the AlN film is highly c-axis oriented on the TC4 substrate. The FWHM values of the AlN film with different thicknesses are shown in Figure 5b. It can be found that the FWHM value of the AlN films decreases with the increase of *t_AlN_*/λ.

**Figure 6:**
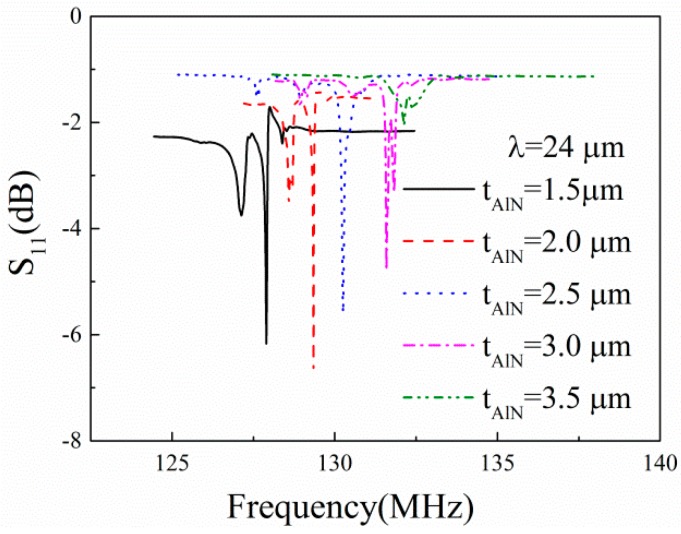
S_11_ parameters of the AlN/TC4 SAW devices. The thicknesses of the AlN films are 1.5 μm, 2.0 μm, 2.5 μm, 3.0 μm, and 3.5 μm, respectively.

Figure 6 shows the measured frequency responses of the one-port SAW resonators on the AlN/TC4 structures. The thickness of the AlN films *t_AlN_* varies from 1.5 μm to 3.5 μm with a step of 0.5 μm. We can observe clear resonance peaks of each SAW device. The resonance frequency increases from 127.90 MHz to 132.09 MHz when the AlN film thickness increases from 1.5 μm to 3.5 μm. Weak spurious peaks occur near the Rayleigh-mode resonance peaks. It is probable that this is due to the defects of the surface electrodes [15].

**Figure 7:**
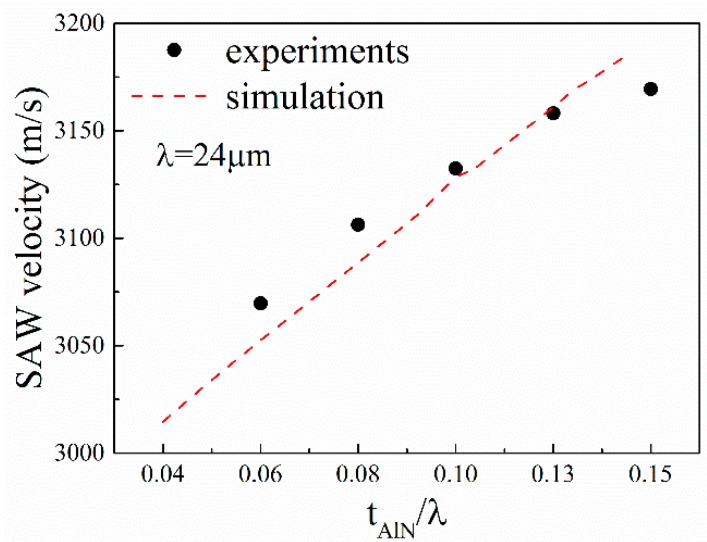
Dependence of experimental (dots) and simulated (dash line) phase velocity on the thickness of AlN films.

From Figure 6, it can be seen that the resonance frequency of the SAW device shifts from low frequency to high frequency with the increase in AlN film thickness. Thus, it can be expected that the surface acoustic wave velocity increases with the increase in AlN film thickness. The calculated SAW velocities with Equation (1) are shown in Figure 7. The simulation results are also presented in Figure 7 for comparison. The SAW velocity is in the range 3060–3170 m/s. One can observe good agreement between the experimental and simulated results.

The prepared SAW devices are characterized in terms of the Q-factor and electromechanical coupling coefficients *k*^2^ to evaluate the performance of the SAW devices. The *k*^2^ of the SAW devices can be deduced from the following equation [16]:

(7) $$k^{2}=\frac{G_{a}}{8f_{0}C_{t}N}$$

where *G_a_* is the radiation conductance, *C_t_* is the capacitance of an IDT pair, *N* represents the number of IDT finger pairs, and *f*_0_ is the resonance frequency. Then, the experimental *k*^2^ can be calculated using Equation (7) with the measured *S*_11_ parameters. The Q-factor is extracted by using the phase slope method [17] and defined as

(8) $$Q=\frac{\omega_{0}}{2}\left|\frac{d\varphi }{d\omega }\right|$$

where ω_0_ is the angular resonance frequency, and Φ is the phase.

**Figure 8:**
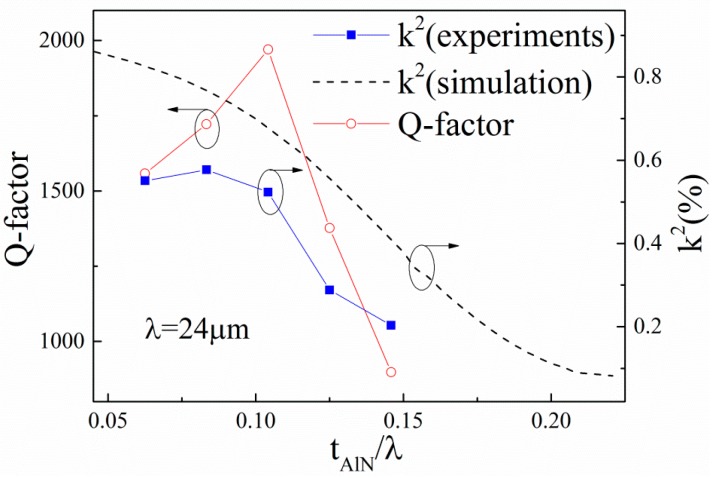
Dependence of the Q-factor and *k*^2^ on the normalized thickness of AlN films.

Figure 8 shows the dependence of the Q-factor and *k*^2^ on the normalized thickness of AlN film. In Figure 8, the maximum *k*^2^ of 0.57% and maximum Q-factor of 1920 are achieved in the AlN/TC4 structure when the *t_AlN_*/λ is 0.0833 and 0.1042, respectively. We can find that the simulated *k*^2^ decreases monotonously with the increase of *t_AlN_*/λ. However, the measured *k*^2^ increases firstly and then decreases with the increase of *t_AlN_*/λ. We think this is because the *k*^2^ is dependent not only on the thickness of AlN films but also on the quality of the AlN films. The FWHM of the AlN film decreased rapidly with the increase of *t_AlN_* when the AlN film was thinner than 2 μm, as shown in Figure 5b, which shows that the *k*^2^ would increase with the increase of *t_AlN_* because the smaller the FWHM, the larger the *k*^2^ [18]. When the *t_AlN_*/λ is greater than 0.1, the *k*^2^ decreases with the *t_AlN_*, which indicates that the thickness effect determines the *k*^2^ and has a negative effect on the performance of the devices. For the same reason, we can find that the dependency of the Q-factor on the *t_AlN_*/λ is similar to that of *k*^2^.

**Figure 9:**
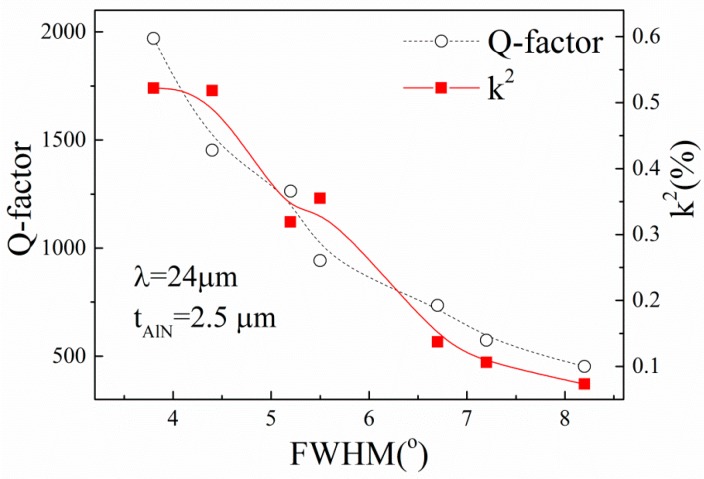
Dependence of Q-factor and *k*^2^ on FWHM where the *t_AlN_* is 2.5 μm. The lines are drawn as a guide for the reader.

From the above results, it can be concluded that the quality of the AlN film is an important factor in affecting the performance of the SAW devices. To explore the effects of the FWHM of the AlN films on the characteristics of the AlN/TC4 SAW devices, we prepared AlN films on the TC4 substrate with different FWHMs by changing the ratio of depositing time at the first and second steps, while the total thickness was fixed to 2.5 μm [14]. The dependence of the Q-factor and *k*^2^ on the FWHM of AlN films is presented in Figure 9.

From Figure 9, it can be found that the Q-factor and *k*^2^ of the SAW devices are strongly dependent on the FWHM of the AlN films. Both Q-factor and *k*^2^ decrease with the increase of the FWHM of the AlN film. The result is agreement with [19]. The dependency of *k*^2^ on FWHM can be explained by the piezoelectricity of the AlN films, which strongly depends on the orientation of the crystalline grain. In general, a small *k*^2^ of the substrates would increase the loss of acoustic energy and lead to a small Q-factor of the realized devices when the devices are fabricated with the same design [20]. This also explained why the performance of the devices, as shown in Figure 8, increases with the increase of *t_AlN_*/λ when *t_AlN_*/λ < 0.1.

**Figure 10:**
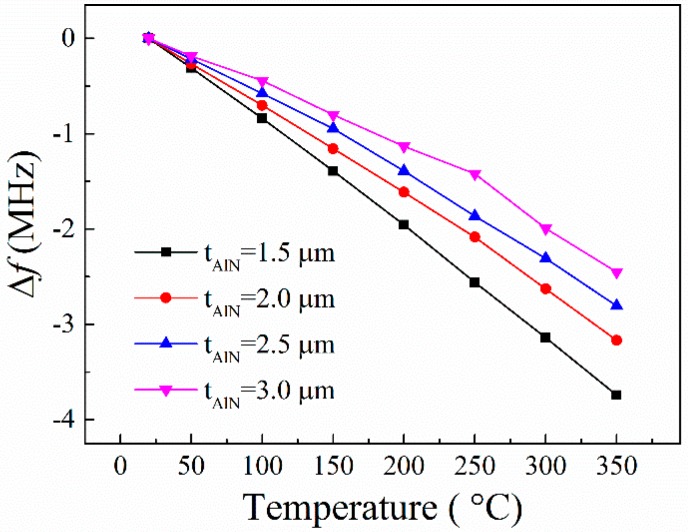
Temperature dependences of the resonance frequency shifts of the prepared AlN/TC4 SAW resonators with different AlN film thicknesses.

The characteristics of the prepared devices were measured at temperatures from room temperature to 350 °C. The dependence of the resonance frequency shifts of the AlN/TC4 SAW resonators on the temperature are plotted in Figure 10. From Figure 10, it can be seen that the devices are characterized by a quasi-linear sensitivity to temperature. Note that the first-order temperature coefficient of frequency (TCF) values of the realized SAW devices increase slightly with the temperature increasing (e.g., the TCF changes from −80 ppm/°C at 20 °C to −101 ppm/°C at 350 °C when the AlN film thickness is 1.5 μm). This result is similar to the previous reports on other AlN film acoustic devices [16,21].

Since the relative AlN thickness *t_AlN_*/λ of the devices is very close to 0.15, the SAW is mainly concerned with the TC4 substrate, as shown in Figure 2a. Thus, the temperature behaviors of the SAW devices are mainly attributed to the TC4 substrate [16]. However, the temperature behaviors of the SAW devices are also affected by the AlN film thickness. The devices with thinner AlN films have larger absolute TCF values than that with thicker AlN films. This is because the AlN has a smaller coefficient of thermal expansion (CTE) than does TC4. When the AlN film thickness increases, more acoustic waves will propagate in the AlN films, leading to a decrease in the effective CTE of the layered AlN/TC4 structure, thus causing a decrease in TCF of the devices [22].

## 5. Conclusions

SAW resonators deposited on an AlN/TC4 structure were investigated in this work. The acoustic wave propagation in AlN/TC4 structure was modeled and simulated by finite element simulations. From the simulation, it was found that there are three regions where the acoustic wave has different propagation modes. Based on the simulation, SAW resonators were fabricated on the AlN/TC4 structure, and the influences of the AlN films were analyzed. We found that the SAW velocity increased with the increase in AlN film thickness. The characteristics of the SAW devices were dependent on the thickness of the AlN film, as well as the quality of the AlN films. The maximum value of Q-factor reached 1970, and the *k*^2^ reached 0.57%. The temperature measurements of the devices show that the AlN/TC4-layered SAW device is characterized by a large and quasi-linear sensitivity to temperature, making it suited for sensing applications in high temperature environments. This work may assist us with a proper design, to fabricate SAW sensors integrated with metal components.

## Figures and Tables

**Table 1:** The material parameters used in the simulation.

Material		AlN [6]	Ti *[7]
density(kg/m^3^)		3260	4510
elastic constant (GPa)	C11	345	162.2
	C12	125	91.8
	C13	120	69
	C33	395	180.6
	C44	118	46.7
	C66	110	35.2
piezoelectric constant (c/m^2^)	e15	−0.48	…
	e31	−0.45	…
	e33	1.55	…
relative permittivity	ε11	9	…
	ε33	11	…

* We use the material parameter of Ti in the simulation because the material parameter of the Ti-6Al-4V (TC4) alloy is close to Ti.
